# The Potential Roles of the Apoptosis-Related Protein PDRG1 in Diapause Embryo Restarting of *Artemia sinica*

**DOI:** 10.3390/ijms19010126

**Published:** 2018-01-02

**Authors:** Wan Zhang, Feng Yao, Hong Zhang, Na Li, Xiangyang Zou, Linlin Sui, Lin Hou

**Affiliations:** 1 College of Life Sciences, Liaoning Normal University, Dalian 116081, China; zhangwan111@yahoo.com (W.Z.); luckyfrog@126.com (F.Y.); hongzhang581@yahoo.com (H.Z.); LiNa812726980@163.com (N.L.); 2 Department of Biochemistry and Molecular Biology, Dalian Medical University, Dalian 116044, China; zouxiangyang@126.com

**Keywords:** *Artemia sinica*, PDRG1, apoptosis, diapauses termination, early embryo development, low temperature stress

## Abstract

High salinity and low temperatures can induce *Artemia sinica* to enter the diapause stage during embryonic development. Diapause embryos stop at the gastrula stage, allowing them to resist apoptosis and regulate cell cycle activity to guarantee normal development after diapause termination. P53 and DNA damage-regulated gene 1 (*pdrg1*) is involved in cellular physiological activities, such as apoptosis, DNA damage repair, cell cycle regulation, and promotion of programmed cell death. However, the role of *pdrg1* in diapause and diapause termination in *A. sinica* remains unknown. Here, the full-length *A. sinica pdrg1* cDNA (*As-pdrg1*) was obtained and found to contain 1119 nucleotides, including a 228 bp open reading frame (ORF), a 233 bp 5′-untranslated region (UTR), and a 658-bp 3′-UTR, which encodes a 75 amino acid protein. In situ hybridization showed no tissue specific expression of *As-pdrg1*. Quantitative real-time PCR and western blotting analyses of *As*-*pdrg1* gene and protein expression showed high levels at 15–20 h of embryo development and a subsequent downward trend. Low temperatures upregulated *As-pdrg1* expression. RNA interference for the *pdrg1* gene in *Artemia* embryos caused significant developmental hysteresis. Thus, PDRG1 plays an important role in diapause termination and cell cycle regulation in early embryonic development of *A. sinica*.

## 1. Introduction

*Artemia sinica* is a small aquatic crustacean living in an environment with a wide variation in temperature and high salt concentration [1,2]. *Artemia* is widely used as the initial feed for the larvae of marine fishes, prawns, and crabs in the aquaculture industry because its nauplii contain abundant proteins and unsaturated fatty acids [3,4]. During embryonic development of *A. sinica,* high salinity, low temperature, light cycle changes, and lack of food can induce *A. sinica* to enter the embryo diapause stage. When the environmental conditions are favorable, it can terminate diapause and restart development from the dormant eggs [5,6]. In process of embryo diapause, dormant cysts of *A. sinica* show strong resistance to long-term drought, cold, hypoxia, and other severe environmental factors. In recent years, there has been wide interest in understanding the causes and molecular mechanisms of diapause and diapause termination of *A. sinica* embryo development. Thus, *A. sinica* has been studied as a good model organism in genetics, development, evolution, and other fields of life science [7,8,9]. Our previous studies suggested that Myeloid differentiation factor 88 (*As*-MYD88) and Late embryogenesis abundant proteins(*As*-LEA) proteins might play vital roles in post-diapause embryonic development and stress tolerance in *A. sinica* [10,11].

P53 and DNA damage control gene 1 (PDRG1) protein were identified as a subunit of the R2TP/prefoldin-like complex, which is involved in the assembly of the RNA polymerase II complex (Pol II) in the cytoplasm of eukaryotic cells [12,13,14]. Previous studies showed that it is involved a variety of cellular physiological activities, such as apoptosis, DNA damage repair, cell cycle regulation and promoting programmed cell death after growth factor starvation [15,16]. Research has shown that the *pdrg1* gene is associated with gastrointestinal degeneration and plays a role in the process of animal organ degradation [15]. That *pdrg1* gene is highly expressed in multiple human malignancies suggests that *PDRG1*, as a high-value novel tumor marker, plays a role in cancer development [17]. The nuclear factor kappa B (NF-κB) pathway is important in immunity, inflammation, development, cell growth, and apoptosis. Some studies have shown that PDRG1 is involved in regulation of the NF-κB pathway [18]. In addition, the stress regulation of *PDRG1* was further studied and found to be induced by genotoxic stress (DNA damage), and its knock down in human colon cancer cells resulted in a significant reduction in tumor cell growth [19].

However, the details of the molecular characteristics and functions of PDRG1 proteins remain unknown in *A. sinica*, especially in diapause embryo termination and early embryo development. In the present study, we cloned the full-length copy DNA (cDNA) of *As*-*pdrg1* and analyzed its expression level in different development periods, temperatures, and salinity stress of *A. sinica* using quantitative real-time PCR (qPCR). In addition, we examined the regulatory relationship among PDRG1, MDM2, P53, and P21 using Western blotting. A small interfering RNA (siRNA) targeting *As-pdrg1* was used to study the function of *As-pdrg1* in embryo diapause termination and early embryonic development. Our aim was to further understand the function and molecular regulation mechanism of PDRG1 with other proteins in the regulation of the cell cycle and apoptosis during postdiapause restarting stages and early embryonic development in *A. sinica*.

## 2. Results

### 2.1. Cloning and Bioinformatic Analysis of As-pdrg1

**Figure 1:**
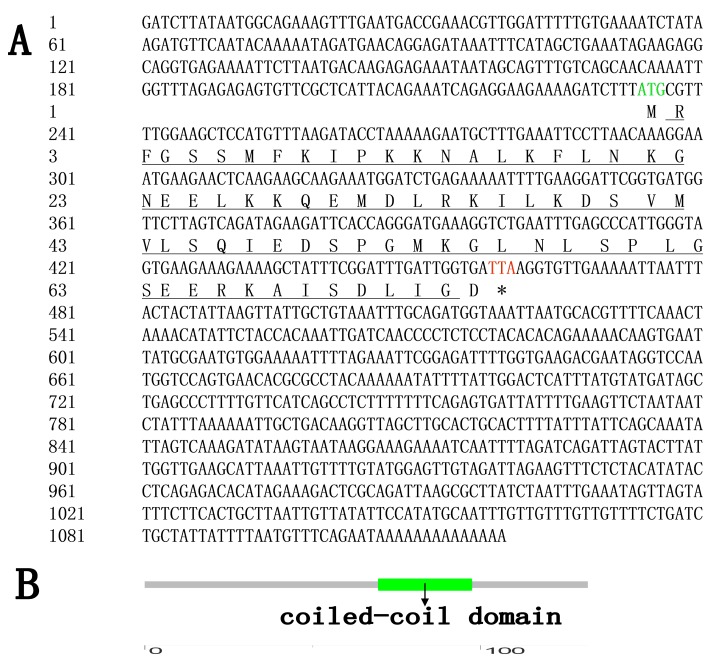
(**A**) Nucleotide sequences and deduced amino acid sequences of the *pdrg1* gene in *A. sinica* (*As-pdrg1*). The numbering of the nucleotide and amino acid sequences is shown on the left. The start codon is indicated in green; the stopcodon is indicated in red. The coiled-coil domain is defined by a straight black line, the asterisk (*) represents the stop codon. (**B**) Results of domain analysis of the putative *As*-PDRG1 protein. The mature putative protein includes a coiled-coil domain (Green).

A 1119 bp full-length cDNA of *As-pdrg1* was obtained (GenBank accession number: MF614121) with an open reading frame (ORF) of 228 bp, and 233 bp 5′-untranslated regions and 658 bp 3′-untranslated regions (Figure 1A). SMART analysis of putative of protein domains showed that the predicted protein contained a coiled-coil domain (Figure 1B). As determined by the Ex-PASy software, the putative PDRG1 protein comprised 75 amino acids, and had a calculated molecular mass of 8.4 kDa, with a pI of 9.35. TMHMM Server 2.0 showed that *As*-PDRG1 has no transmembrane region. According to Protscale analysis *As*-PDRG1 is mostly hydrophilic. No signal peptide was identified, which indicated that *As*-PDRG1 is a non-secreted protein. The online software PSORTII predicted that *As*-PDRG1 might be located in the nucleus, mitochondria, and cytoplasm. NetPhos 2.0 Server analysis indicated that there are eight possible serine phosphorylation sites (score > 0.5) (Table 1).

**Figure 2:**
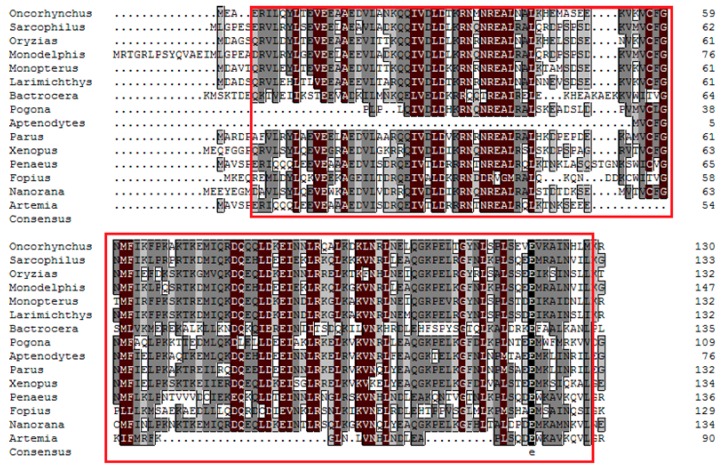
Phylogenetic tree of aligned amino-acid sequences of *As-pdrg1* and 14 other species. A neighbor joining phylogenetic tree was constructed based on the amino acid sequences of *As-pdrg1* from this study and 14 other species from GenBank using the sequence analysis tool MEGA 6.0. The sequences and corresponding GenBank accession numbers are as follows: *Monodelphis domestica*, XP_001362684.2; *Sarcophilus harrisii*, XP_003757064.1; *Oryzias latipes*, XP_004068945.1; *Monopterus albus*, XP_020473939.1; *Oncorhynchus kisutch*, XP_020330168.1; *Larimichthys crocea*, XP_010742575.1; *Aptenodytes forsteri*, XP_019328545.1; *Parus major*, XP_015503186.1; *Xenopus tropicalis*, NP_001015688.1; *Nanorana parkeri*, XP_018411923.1; *Fopius arisanus*, JAG81456.1; *Penaeus monodon*, APL97140.1; *Bactrocera cucurbitae*, XM_021527437.1; *Crocodylus porosus*, XM_019539779.1; *Artemia sinica*, MF614121. The red box indicates *As*-PDRG1. Identical amino-acid residues are indicated by black and reddish brown boxes. Less conserved residues are indicated by gray boxes, whereas somewhat similar residues are indicated by pale gray boxes. The pdrg1 N-terminal and C-terminal domains are indicated by red boxes. For interpretation of the references to color in this figure legend, the reader is referred to the web version of this article.

**Figure 3:**
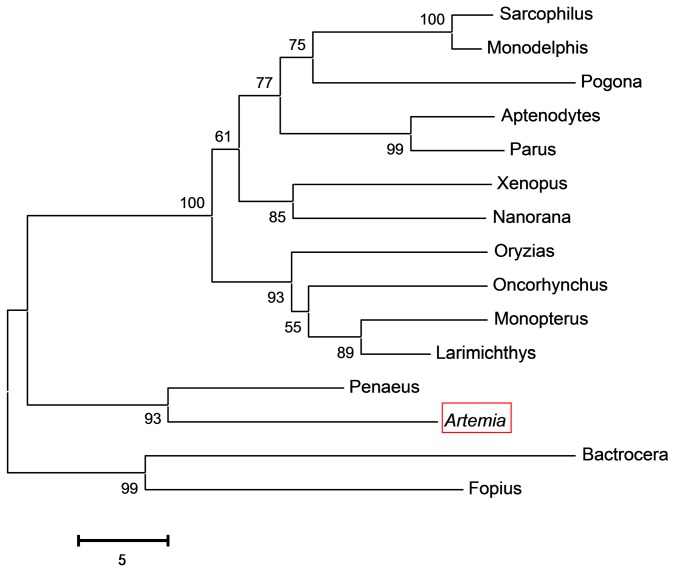
Alignment of *As*-PDRG1 and known PDRG1 sequences from 15 other species. The sequences and their accession numbers are the same as those detailed in the legend of Figure 2. A red box indicates *As*-PDRG1.

Alignment of *As*-PDRG1 and known PDRG1 sequences from 15 other species revealed the homology of *As*-PDRG1 with other PDRG1 protein sequences. *As-pdrg1* shares 75% similarity with PDRG1 of *Penaeus monodon* (Figure 2). To evaluate the evolutionary relationships among PDRG1 homologs, a phylogenetic tree composed of 15 species and *A. sinica* with 1000 bootstrap replications was constructed using Clustal X2.0 and MEGA 6.0. Analysis of the phylogenetic tree (Figure 3) showed that there are three main clusters. The first cluster contains higher vertebrates (*Monodelphis domestica*), amphibians (*Xenopus tropicalis*), birds (*Aptenodytes forsteri*), and fish (*Oryzias latipes*). The second cluster comprises arthropods (*Penaeus monodon* and *Artemia*) and the third cluster comprises insects (*Fopius arisanus*). The relationships displayed in the phylogenetic tree are consistent with *A. sinica*’s taxonomic classification.

### 2.2. Expression Level of As-pdrg1 at Different Developmental Stages and under Different Stresses

**Figure 4:**
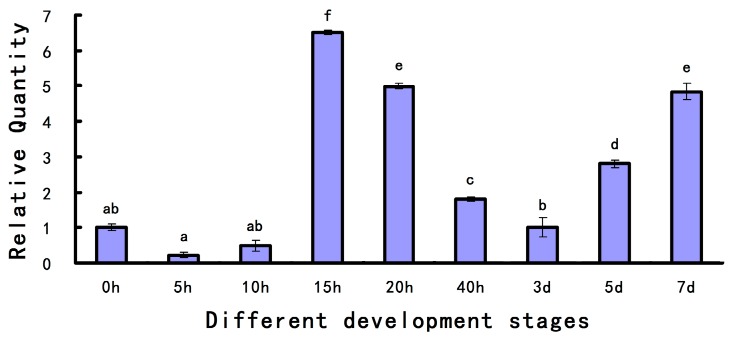
Quantitative real-time PCR (qPCR) analysis of *As-pdrg1* expression during different developmental stages of *A. sinica*. The messenger RNA (mRNA) expression levels of *As-pdrg1* and β-actin were measured at various time points during development, and data are presented as means ± SD of triplicate experiments. The development stage at 0 h was set as the control group. The *x*-axis indicates the developmental stage (0 h–7 days); and the *y*-axis indicates the expression level relative to expression at 0 h. Significant differences between developmental stages (*p* < 0.05) were analyzed by one-way analysis of variance (ANOVA) and are indicated with letters (a–f).

Analysis with qPCR was performed to detect the expression levels of *As*-*pdrg1* during different stages of embryo development. The data showed that a low level of *As-pdrg1* messenger RNA (mRNA) from 0 to 10 h, which then increased to reach a peak at 15 h. The expression levels decreased from 15 h to 3 days, at which point the expression level of *As-pdrg1* was about one third of the level at 15 h. After 3 days, the expression level started to increase; the expression levels at 7 days and 20 h were almost the same (Figure 4).

**Figure 5:**
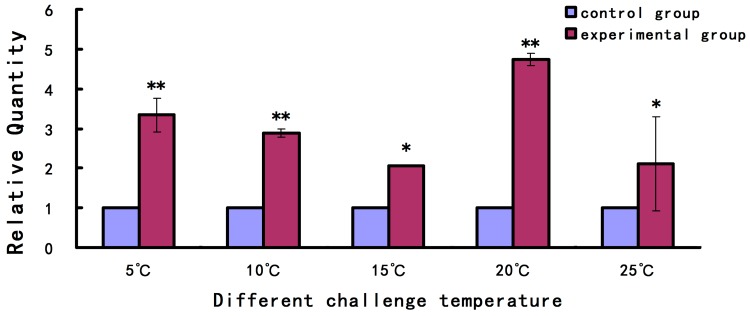
Quantitative real-time PCR (qPCR) analysis of *As-pdrg1* expression in response to temperature stress. The mRNA expression levels of *As-pdrg1* were measured after challenge with five different temperature concentrations. The 30 °C treatment condition served as the control. Data are presented as the means ± SD of triplicate experiments. Highly significant differences between the experimental and control groups are indicated with ** *p* < 0.01, while significant difference are indicated with * 0.01 < *p* < 0.05.

The expression level of *As-pdrg1* under different salinity and temperature stress conditions were analyzed by qPCR. The results showed that the expression level of *As-pdrg1* was higher at lower temperatures than that under the control temperature (30 °C), the expression level of *As-pdrg1* reached a peak at 20 °C, being about four times that of the control group (Figure 5).

### 2.3. Expression Location of As-pdrg1

**Figure 6:**
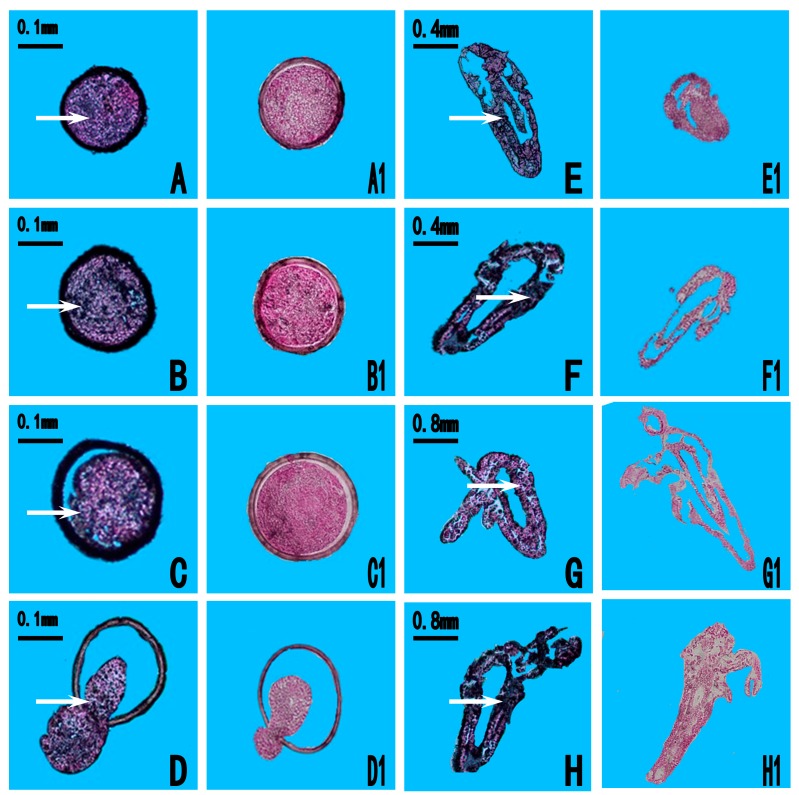
In situ hybridization analysis of *As-pdrg1* expression during different developmental stages of *Artemia sinica*. (**A**–**H**) experimental groups, (**A1**–**H1**) control groups. (**A**) 0 h, gastrula stage of Artemia cysts; (**B**–**D**) 5 h, 10 h and 15 h, embryonic stage; (**E**,**F**) 20 h and 40 h, nauplius stage; (**G**) 3 days, metanauplius larva stage; (**H**) 5 days metanauplius stage. Arrows indicate positive signal regions.

In situ hybridization was performed to determine the spatial expression pattern of *As-pdrg1* at different embryo developmental stages of *A. sinica* (Figure 6). Compared with the control group, *As-pdrg1* signals were observed throughout almost the whole embryo during the prenauplius stage (0 h, 5 h, and 10 h; Figure 6A–C). Expression gradually extended from head to the tail in the umbrella stage at 15 h (Figure 6D). At 20 h, *As-pdrg1* mRNA was detected significantly in the whole larval body after the nauplius had hatched (Figure 6E). At 40 h *As-pdrg1* mRNA was observed throughout almost the entire body (Figure 6F). At 3 days and 5 days, the body shape and external appendages begin to develop, and *As-pdrg1* was detected in the region of the enteron and the external appendages of the polypide (Figure 6G,H). No signal was detected in the control group under the same conditions (Figure 6A1–H1).

### 2.4. Purification and Expression of As-PDRG1 Protein

**Figure 7:**
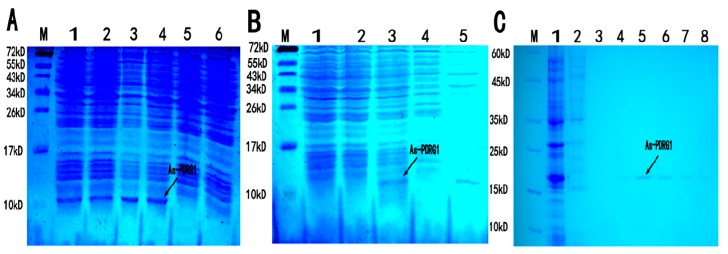
(**A**) Expression of *As*-PDRG1 recombinant protein. M: Protein markers from 10 to 72 kDa. Lanes 1–4 show the expression of the *As*-PDRG1 recombinant protein from four induction treatments. (1 mM isopropyl β-d-1-thiogalactopyranoside (IPTG) at 37 °C, 1 mM IPTG at 30 °C, 0.25 mM IPTG at 37 °C, and 0.25 mM IPTG at 30 °C, respectively). The arrow shows the position of the expressed recombinant protein. Lane 5: Total proteins from non-induced cells. Lane 6: Total proteins from induced cells harboring vector pET-30a (control). (**B**) Detection of soluble *As*-PDRG1 recombinant protein (control). Lane 1: Total proteins from uninduced cells. Lane 2: Total proteins from induced cells harboring vector pET-30a (control). Lane 3: Total *As*-PDRG1 recombinant protein. Lane 4: Soluble fraction of the lysate from induced cells harboring pET-30a-PDRG1. Lane 5: insoluble fraction of the lysate from induced cells harboring pET-30a-PDRG1. (**C**) Purification of recombinant *As*-PDRG1 protein. M: Protein markers from 10 to 60 kDa. Lane 1: Unpurified, induced *As*-PDRG1 recombinant protein. Lane 2: Flow-through eluate of total proteins. Lanes 3–8: Column elution with elutant containing 5 mM, 10 mM, 20 mM, 40 mM, 60 mM and 80 mM imidazole, respectively.

A 228 bp fragment of the ORF was obtained by digestion with restriction enzymes *Eco*RI and *Xho*I, which encoded the putative PDRG1 protein of 75 amino acids. The predicted molecular mass was about 8.4 kDa. The PDRG1 protein isoelectric point was 9.35. It showed no significant difference in expression quantity under the four different induction conditions (Figure 7A), therefore, we adopted a condition of 1 mM IPTG at 37 °C for further research. Sodium dodecyl sulfate polyacrylamide gel electrophoresis (SDS-PAGE) detected the molecular mass of the recombinant protein as 11 kDa, which was consistent with the predicted size. We identified that the PDRG1 protein was expressed in an inclusion body form (Figure 7B). After denaturation in 20 M urea, purification, and dialysis, a relatively pure protein was obtained (Figure 7C).

### 2.5. Expression Pattern of As-PDRG1 Tested by Western Blotting in Early Embryo Development and to Stress

**Figure 8:**
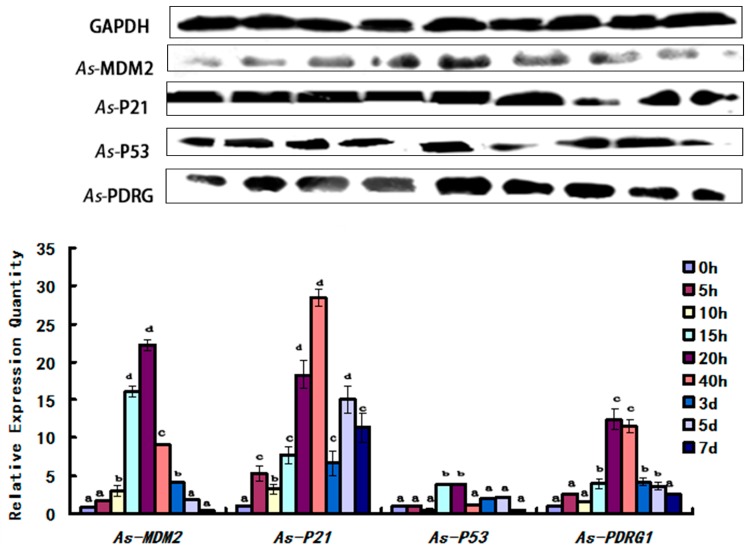
Western blot showing the abundances of *As*-MDM2, *As*-P21, *As*-P53, As-PDRG1 proteins at different developmental stages of *A. sinica*. The band intensities of these proteins were normalized against that of GAPDH. The expression of these proteins at 0 h was used as the reference; letters (a–d) indicate statistically significant differences (*p* < 0.05).

The experimental data from different embryo development stages showed that the amount of *As*-PDRG1 in *A. sinica* increased from 0 to 20 h, and reached its peak at 20 h. Subsequently, it began to decline. Its lowest level was at 7 days. The cell cycle regulatory proteins (*As*-MDM2, *As*-P21, *As*-P53) showed similar trends to *As*-PDRG1. Meanwhile, the level of P53 was less than that of PDRG1 (Figure 8).

**Figure 9:**
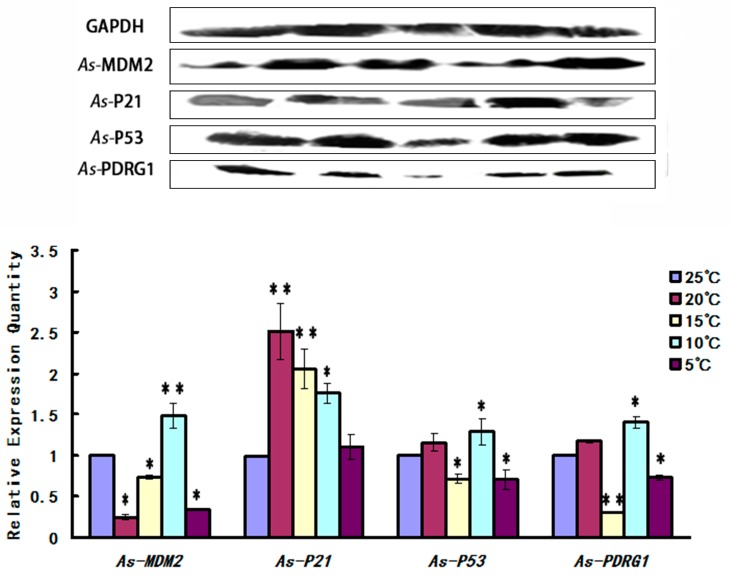
Western blot showing the abundances of *As*-MDM2, *As*-P21, *As*-P53, and *As*-PDRG1 proteins in response to temperature stress in *A. sinica*. The band intensities of these proteins were normalized against that of GAPDH. Expressions of the proteins at 25 °C served as the control. Statistically significant differences are indicated with ** *p* < 0.01, while * represents 0.01 < *p* < 0.05.

In the temperature stress experiment, the experimental data showed that as the temperature decreased from 25 to 15 °C, the *As*-PDRG1 protein level showed a downward trend and the lowest value occurred at 15 °C. As the temperature continued to decrease, the level of *As*-PDRG1 protein increased. The cell cycle regulatory proteins (*As*-MDM2, *As*-P53) showed similar trends to *As*-PDRG1. Meanwhile, the cell cycle regulatory protein *As*-P21 showed a sharp rise from 25 to 20 °C; the maximum level was observed at 20 °C. Thereafter, with decreasing temperature the level of *As*-P21 also decreased (Figure 9).

### 2.6. Short Interfering RNA (siRNA) for Knockdown of As-PDRG1

**Figure 10:**
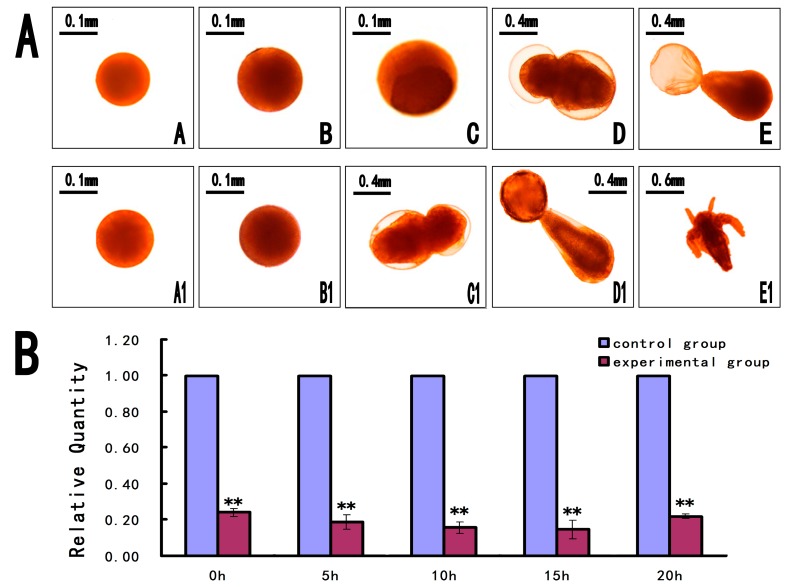
(**A**) The relative level of *As-pdrg1* mRNA expression in larvae soaked with double-stranded RNAs (dsRNAs) for different times. *Pdrg1*-RNAi depleted the expression of *As-pdrg1* at different developmental stages from 0 to 20 h in *A. sinica*. A–E represents experimental groups treated with *pdrg1*-RNAi and A1–E1 represent the control group. (**B**) Shows the reverse transcription PCR (RT-PCR) results for the corresponding period. Statistically significant differences are indicated with ** *p* < 0.01.

To explore the function of *As-pdrg1* during the early embryo development of *A. sinica*, we knocked down *As-pdrg1* expression using an siRNA. After knockdown of *As-pdrg1* by the siRNA, phenotypic observation showed almost no difference between the experimental and control groups from 0 to 5 h. Abnormal individuals appeared at the nauplius stage (10–20 h): growth and movement of the individuals in the experimental group were slow compared with the control (Figure 10A). The death and deformation rates both increased. The mortality of the experimental group was 40.25% compared with 25.68% for the control group. Reverse transcription PCR (RT-PCR) analysis showed that the expression level of the *As-pdrg1* mRNA in the experimental group was lower than that in the control during the five different periods (0 h, 5 h, 10 h, 15 h, and 20 h; Figure 10B).

To explore the function of *As-pdrg1* during the early embryo development of *A. sinica*, we knocked down *As-pdrg1* expression using an siRNA. After knockdown of *As-pdrg1* by the siRNA, phenotypic observation showed almost no difference between the experimental and control groups from 0 to 5 h. Abnormal individuals appeared at the nauplius stage (10–20 h): growth and movement of the individuals in the experimental group were slow compared with the control (Figure 10A). The death and deformation rates both increased. The mortality of the experimental group was 40.25% compared with 25.68% for the control group. Reverse transcription PCR (RT-PCR) analysis showed that the expression level of the *As-pdrg1* mRNA in the experimental group was lower than that in the control during the five different periods (0 h, 5 h, 10 h, 15 h, and 20 h; Figure 10B).

## 3. Discussion

In this study, we cloned the full-length cDNA of *As-pdrg1* and noted that it encodes a putative protein of 75 amino acids (8.4 kDa), which is smaller than the 133 amino acids of both human and mouse PDRG1 proteins. However, other species also have shorter PDRG1 proteins, such as *Aptenodytes forsteri* (76 aa) and *Pogona vitticeps* (109 aa). Although the short type PDRG1 is rare, the alignment results showed that the deduced amino acid sequence QEIVDLDTKRNQNREALRL (30–48 aa) of *As*-PDRG1 shares high homology with PDRG1 proteins from other species. However, the function difference between the short and long types of PDRG1 is unclear, and further research is needed to determine the significance of this difference [20]. As an important protein for the cell cycle, *As*-PDRG1 is ubiquitously expressed in the nucleus, mitochondria, and cytoplasm. *As*-PDRG1 has no transmembrane region and it was mostly hydrophilic, according to the Protscale analysis. No signal peptide was identified, which indicated that *As*-PDRG1 is a non-secreted protein. These are similar to previous studies of the *pdrg1* gene from the sea cucumber [15,16]. Thus, *pdrg1* in *A. sinica* is similar to that in other invertebrates. According to the results of ISH, the *As-pdrg1* gene is transcribed in different tissues and organs of *A. sinica*. Positive signals were detected in the early embryo and prenauplius stage (0–10 h). In the subsequent embryonic development process, *As-pdrg1* was detected in almost in all parts, which suggested that it plays diverse roles in embryonic development and diapause termination of *A. sinica.*

Previous studies have shown that PDRG1 is a multifunctional protein that is involved a variety of cellular physiological activities, such as apoptosis, DNA damage repair, cell cycle regulation, and preventing programmed cell death after growth factor starvation [15,16,17]. The results of both qPCR and Western blot demonstrated that the mRNA and protein expression of *As*-PDRG1 showed the same increasing trend at the early developmental stage from 0 to 20 h. During the initial developmental stage of *A. sinica*, the cysts in diapause were activated and gradually increased. From 0 to 20 h, the embryos progressed from cysts to nauplii. The embryonic cells may experience the rapid cell division and proliferation that are necessary for embryonic activities. Along with cell division, apoptosis occurs. The increase in the mRNA and protein expression levels of *As*-PDRG1 could promote apoptosis to remove the unwanted, damaged, and dangerous cells during development, and to maintain homeostasis, which suggested that *As*-PDRG1 plays an important role in diapause termination and early embryonic development of *A. sinica*. Thereafter, western blotting showed that *As*-PDRG1 began to decrease until 7 days. However, the *As-pdrg1* mRNA expression reduced until 3 days, and then gradually increased. The decrease in *As-pdrg1* expression could be caused by p53 expression. Western blotting showed that p53 increased from 15 to 20 h. Previous studies indicated that p53 negatively regulates *PDRG1* at the transcriptional level. Meanwhile, we also detected the expression of *As*-PDRG1 under low temperatures. The qPCR results showed that the expression level of *As*-pdrg1 increased gradually as the temperature decreased. Western blotting showed that as the temperature fell from 25 to 15 °C, the *As*-PDRG1 protein expression showed a downward trend; the lowest value occurs at 15 °C. These results are consistent with the hypothesized function for *As*-PDRG1. The qPCR result agreed with the results of two other studies concerning genes relevant to the cell cycle in *A. sinica* [21,22].

To study the function of *As*-PDRG1, the P53 and related proteins (MDM2, P21) levels were analyzed by Western blotting. The results showed that *As*-MDM2, *As*-P21, and *As*-P53 showed similar increasing trends to *As*-PDRG1 from 15 to 40 h. Although *As*-PDRG1 expression decreased under P53 negative regulation, *As*-PDRG1 showed high expression at 20 and 40 h. The increased protein level occurred in a P53 independent manner [23,24,25]. It is possible that strong positive regulation by certain other genes could override the negative effect of P53 on PDRG1. This phenomenon was similar to the results of Luo [16]. In their study, the *PDRG1* promoter was shown to harbor Oct1-binding sites and Oct1 strongly upregulated *PDRG1* expression following genotoxic stress. Further research on the genetic regulation of *As-pdrg1* is required.

PDRG1 has functions such as DNA damage repair; therefore, we observed the expression of *As-pdrg1* under low temperatures. *As-pdrg1* showed high expression under low temperatures; however, at the protein level, this phenomenon was not so obvious. The results suggested that *As*-PDRG1 is likely involved in DNA damage repair caused by low temperature. However, *As*-PDRG1 is likely to be involved in more complex regulatory mechanisms, resulting changes in its protein expression and inconsistent in RNA levels. The mechanisms of this regulation are unknown. This experiment also verified that the cell cycle regulators *As*-MDM2 and *As*-P21 belong to the P53 signaling pathway. In addition, we confirmed that MDM2 and P53 are in negative feedback regulation [26]. P21 protein is downstream protein of P53; however, expression levels of P53 and P21 showed obviously different trend during 3–7 days development stages from *Artemia sinica*. It is reason may be exist a complicated regulatory mechanism to affect the expression of P21 and P53, in process of regulation, ATM protein can directly affect the expression level of P21 without P53 regulation, leading to changes of expression level [27,28,29].

To determine the function of the protein, we carried out siRNA knockdown experiments. RNA interference reduces transcription, thereby reducing the levels of the target protein, which may have phenotypic effects. In our *pdrg1* knockdown experiments, increased mortality was observed in larvae expressing the siRNA targeting *As-pdrg1*. Surviving individuals not only moved slower than the wild-type, but also developed more slowly. This was similar to a previous study, in which knockout of *pdrg1* slowed cell growth [15]. Therefore, the siRNA results suggested that *pdrg1* plays an important role in early embryonic development.

## 4. Materials and Methods

### 4.1. Sample Reparation

The cysts of *A. sinica* were harvested from the salt lake of Yuncheng, Shanxi Province (China) and stored at −20 °C in the dark until use. The cysts were hatched in filtered seawater (salinity 28‰) under laboratory conditions: 30 °C, at an illumination intensity of 1000 lx, according to the procedure described by Cheng et al. [30].

The development of *A. sinica* consists of five main stages: the embryo, nauplius, metanauplius, pseudoadult, and the adult stages. In this experiment, 0–10 h corresponded to the cyst stage; 15–20 h corresponded to the nauplius stage; 40 h corresponded to the metanauplius stage; 3 days and 5 days corresponded to the pseudoadult stage; and after 5 days corresponded to the adult stage. Animal samples of roughly 0.5 g were collected at each time point (0 h, 5 h, 10 h, 15 h, 20 h and 40 h; 3 days, 5 days and 7 days, adult) at different periods of development for subsequent experiments. For the salinity stress condition, *A. sinica* nauplii (20 h) were maintained at 30 °C in 28‰ salinity natural seawater for 44 h and used as the control group. *A. sinica* at the same stage (20 h) were then treated with high salinity (50‰, 100‰, 150‰, and 200‰). The low temperature tolerance and salinity stress conditions used the same control group. While *A. sinica* at the same stage (20 h) were held at 25, 20, 15, 10, or 5 °C for the low temperature stress test [31,32,33,34].

### 4.2. Cloning the Full-Length pdrg1 cDNA

Total RNA from the different developmental periods was extracted using the TRIZOL-A+ reagent (Takara, Dalian, China), according to the manufacturer’s instructions. The RNA was then reverse transcribed into cDNA using an oligo(dT) primer and MMLV reverse transcriptase (Takara), following the manufacturer’s instructions. Specific primers (*As*-*pdrg*1F, *As*-*pdrg1*R, Table 2) were designed using primer Premier 5.0 based on the partial sequence of *Artemia franciscana pdrg1* and synthesized by GENEray (Shanghai, China). The PCR reaction conditions were as follows: initial incubation at 94 °C for 5 min; followed by 35 cycles of denaturation at 94 °C for 30 s, annealing at 56 °C for 30 s, and elongation at 72 °C for 1 min; and a final incubation at 72 °C for 10 min. PCR products were separated on 1.0% agarose/Tris-Acetate-EDTA (TAE) gels and sequenced by TsingKe (Beijing, China). This produced a 350 bp expressed sequence tag (EST) sequence of *As*-*pdrg1*. To obtain the full-length cDNA sequence of *As*-*pdrg1*, rapid amplification of cDNA ends (RACE) was performed. The 3′ and 5′ rapid-amplification of cDNA ends (RACE) reactions were carried out using a SMART™ RACE cDNA Amplification Kit amplification kit (Clontech, Dalian, China). All the reaction processes of the 3′ and 5′ RACE were carried out according to the manufacturer’s instructions. The primers for 3′ RACE (3′ *As*-*pdrg1*, Table 2) and 5′ RACE (5′ *As*-*pdrg1*, Table 2) were designed based on the amplified 350 bp gene fragment of *As*-*pdrg1* mentioned above. The RACE PCR products were purified using a Gel Extraction Kit (Trans, Beijing, China) and cloned into the pEasy transformed into *Escherichia coli* strain T1, and then sequenced by TsingKe. The 3′ and 5′ termination fragments were spliced together using DNA Man 8.0 (Lynnon Biosoft, San Ramon, CA, USA) to obtain the full-length *As*-*pdrg1* cDNA. The full-length *pdrg1* nucleotide sequence was submitted to GenBank with the accession number MF614121.

### 4.3. Quantitative Real-Time PCR (qPCR)

#### 4.3.1. Expression of *As-pdrg1* at Different Developmental Stages

Samples of *A. sinica* were collected at different growth periods (0 h, 5 h, 10 h, 15 h, 20 h, and 40 h; 3 days, 5 days, and 7 days) and the total RNA was extracted as templates for cDNA synthesis using a Two-step Reverse Transcription Kit (Takara), following the manufacturer’s instructions. The primers for real-time PCR (RT-*pdrg*1F, RT-*pdrg*1F; Table 2) were synthesized by TsingKe. The *A. sinica* β-actin gene (β-actin-F, β-actin-F; Table 2) was used as a normalization control for each RNA sample [19,20]. Real-time PCR was performed in triplicate for each sample using SYBR^®^ Premix Ex Taq™ (Takara). Each reaction (25 μL) contained 0.5 μL of each primer, 12.5 μL of Master SYBR Green I (Takara) mix, 2 μL of cDNA template, and water to a final volume of 25 μL. The qPCR reaction conditions were as follows: 95 °C for 30 s; followed by 40 cycles each of 95 °C for 5 s, 56 °C for 30 s, 95 °C for 15 s, 60 °C for 30 s, and 95 °C for 15 s. At the end of each reaction, the experimental data were gathered from the three parallel reactions [5]. Subsequently, the gene expression data were analyzed by Thermal Cycler Dice Real Time system software (Takara), and quantified by the comparative cycle threshold (*C*t) method (*C*t^2−ΔΔ^ method), based on the *C*t values for both *As-pdrg1* and β-actin, to calculate the relative fold increase [35,36,37]. The statistical significance of any change was analyzed using the least square difference (LSD) *t*-test in SPSS 16.0. The significance threshold was *p* < 0.05.

#### 4.3.2. Expression of *As*-*pdrg1* in Response to Stress

For the temperature challenge assay, we used the method shown in Section 4.1. *A. sinica* nauplii (20 h) were maintained at 30 °C in 28‰ salinity natural seawater for 44 h and used as the control group. *A. sinica* at the same stage (20 h) were held at 25, 20, 15, 10, or 5 °C for the low temperature stress test in 28‰ salinity natural seawater for 24 h. Total RNA was extracted from the six groups, and the RNA samples were reverse transcribed into cDNA templates.

For the salinity challenge assay, we used the method shown in Section 4.1. The sample reared for 44 h in natural seawater (salinity 28‰) served as the control. The experimental groups were incubated in salinities of 50, 100, 150, and 200‰ for the high salinity stress test in 28‰ salinity natural seawater for 24 h. RNA was extracted from each salinity sample and reverse transcribed into cDNA for qPCR, which was carried out as described in Section 4.3.1.

### 4.4. Construction, Expression, and Purification of pET-30a PDRG1 Protein

The full-length ORF of *As*-*pdrg1* was amplified using primers that added *Eco*RI and *Xho*I sites to the 5′ and 3′ ends. The primers (ORF-*pdrg1*F, ORF-*pdrg1*F, Table 2) were designed using Primer Premier 5.0 software. The PCR products were cloned into the pMD19T vector. PMD19T-*pdrg1* and pET30a were both digested with *Eco*RI and *Xho*I and then ligated together using T4 DNA ligase (Takara) at 16 °C overnight. TsingKe then sequenced the recombinant plasmid, pET-30a-*pdrg1*.

### 4.5. Expression and Purification of the Recombinant Protein from Escherichia Coli

To facilitate the overexpression of PDRG1, the recombinant expression plasmid pET-30a-*pdrg1* was transformed into *E. coli* BL21 (DE3). Four different conditions were used to induce the expression of the fusion protein: 1 mM isopropyl β-d-1-thiogalactopyranoside (IPTG) for 3 h at 37 °C, 1 mM IPTG for 3 h at 30 °C, 0.25 mM IPTG for 3 h at 37 °C, and 0.25 mM IPTG for 3 h at 30 °C. Cells were collected and washed with phosphate buffered saline (PBS) three times. The cells were collected by centrifugation at 2800× *g* for 5 min, the supernatants were discarded, and the pellets were washed twice with PBS, and then resuspended in PBS. The samples were mixed with 5 × SDS-PAGE loading buffer, at a ratio of 4:1, and boiled for 10 min.

The recombinant protein was expressed in a 500 mL culture of *E. coli* BL21 (DE3) and induced with 1 mM IPTG at 37 °C for 3 h. The cells were collected by centrifugation at 4 °C, and washed twice with 1× PBS. The pellets were resuspended in equilibration buffer, and then lysed by ultrasonication. Subsequently, the inclusion bodies were washed twice with 6.057 g/L Tris containing 5.85 g/L NaCl and 120.12 g/L Urea and Ethylene Diamine Tetraacetic Acid (EDTA) 0.2922 g/L, followed by dissolution in a solution comprising Tris 12.114 g/L, 29.25 g/L NaCl, 480.48 g/L Urea, and 0.68077 g/L imidazole. The lysate was loaded onto an Ni-NTA HisTrap™ HP crude column (GE Healthcare, Little Chalfont, UK) and then eluted using elution buffer at different imidazole concentrations (5, 10, 20, 40, 60, 80, and 100 mM). The terminal products were dialyzed into 20 mM Tris-HCl and then the samples were mixed with 5× SDS-PAGE loading buffer, at a ratio of 4:1, and boiled for 10 min. A small amount of the samples were examined by SDS-PAGE on a 15% gel.

### 4.6. In Situ Hybridization (ISH) Assay

Templates for the RNA probes were prepared by PCR with a pair of specific primers (ISH-*pdrg1*F, ISH-*pdrg1*F, Table 2) designed based on the full-length sequence of *As*-*pdrg1*. The PCR product (about 396 bp) was purified on an agarose gel and cloned into the pGM-T vector (Tiangen, China), which added the T7 and SP6 polymerase binding sites. RNA probes were labeled with digoxigenin (DIG) using a DIG RNA labeling kit (SP6/T7, Roche, Indianapolis, IN, USA), according to the manufacturer’s instructions. Samples were then collected at different developmental stages (0 h, 5 h, 10 h, 15 h, 20 h and 40 h; 3 days and 5 days). The 0 h, 5 h, and 10 h samples were first completely decapsulated with 50% NaClO. The samples were washed with diethylpyrocarbonate (DEPC) quickly, and fixed in freshly prepared 4% paraformaldehyde solution in 100 m/mol PBS (pH 7.4) at 4 °C for 4–8 h. Subsequently, the samples were taken through an ascending ethanol concentration series (30%, 50%, and 70%), and then stored in 70% EtOH. The samples were then dehydrated with concentrated gradient of ethanol (80–90%) for 1 h each, and then dehydrated with ethanol for 1 h, twice. To make them transparent, the samples were treated with xylene:ethanol (1:1) ratio for 15 min, and then with xylene for 10 min, followed by xylene:paraffin wax (1:1) for 30 min at 60 °C embedded organization.

The embedded tissue was cut into 8 μm-thick slices using a microtome and placed on 0.1% DEPC water-sterilized glass slides treated with polylysine on a 42 °C stage for 1 h, incubated at 50 °C overnight and then dried and sealed at 4 °C. After dewaxing the samples, they were incubated sequentially in 1× PBS, 0.3% TritonX-100, RNase-free ProK, 4% paraformaldehyde (pH 7.4), washing buffer, blocking solution, antibody solution, and detection buffer (pH 9.5). Prehybridization of each sample was then performed at 52 °C for 2 h in prehybridization buffer containing 50% deionized formamide (*v*/*v*), 1× Denhardt’s solution, 5× saline-sodium citrate (SSC), and 5 mg/mL salmon sperm DNA at 50 °C overnight. Subsequently, a DIG nucleic acid detection kit (Roche) was used to detect the signal. Finally, nuclear fast red was used for counterstaining and signals were detected under a microscope.

### 4.7. Western Blotting

#### 4.7.1. Protein Production of *As*-PDRG1 in Different Stages of Early Embryo Development

Total proteins were extracted from each sample (0 h, 5 h, 10 h, 15 h, 20 h, and 40 h; 3 days, 5 days, 7 days) using Radioimmunoprecipitation (RIPA) Lysis Buffer (Solarbio, Beijing, China) and quantified using the Bradford method [38,39,40] with a BCA Kit (Solarbio), according to the manufacturer’s instructions. Samples (70 μg) were subjected to fractionation by 15% or 12% SDS-polyacrylamide gels and transferred to polyvinylidene fluoride (PVDF) membranes. The membranes were blocked with 5% non-fat powdered milk (Sangon, Shanghai, China) over 1 h at room temperature. The membranes were then placed in sealed plastic bags containing diluted (in PBS with Tween 20 (PBST)) rabbit anti-*As*-PDRG1 polyclonal antibodies (1:200) and anti-GAPDH antibodies (1:1000), and incubated overnight at 4 °C. The membranes were washed with PBST (3 × 10 min), incubated with horseradish peroxidase (HRP)-conjugated goat anti-rabbit IgG and goat anti-Mouse IgG anti-bodies at 37 °C for 1 h, followed by washing with PBST (3 × 10 min). Immunoreactive proteins were visualized using the ECL reagent (Advansta, Menlo Park, CA, USA) and the blots were exposed to an X-ray film in the darkroom. Gray scale analysis using the Image J software (available online: https://imagej.nih.gov) was used to compare the density (equivalent to the intensity) of the bands on the blots and the data were then converted into column charts. The expression intensities of *As*-PDRG1 specific bands were normalized against those of the GAPDH bands.

#### 4.7.2. Expression of *As*-PDRG1 under Low Temperature

For the temperature-challenge assay, cysts were reared for 20 h at 25 °C. The temperature was then reduced to 20 °C, 15 °C, 10 °C, and 5 °C and incubation continued for 24 h. The method was shown in Section 4.7.1. It was used to obtain total proteins. Western blotting was performed to analyze the levels of *As*-PDRG1 in response to low temperature, with GAPDH as the control. All antibodies were bought from Proteintech (Wuhan, China).

### 4.8. Short Interfering RNA (siRNA) Assay

The RNA oligonucleotides were designed using online software from the full-length cDNA of *As*-*pdrg1* and synthesized by Takara (Sense siRNA, Anti siRNA; Table 2). The 0 h samples were exuviated using 50% NaClO. Subsequently, the samples were suspended in electroporation buffer, 2 mM dsRNAs (siRNAs) were added, and the samples (1.3 mL in electroporation buffer and dsRNA) were added to an electroporation cuvette, and placed into the electroporator. The samples were electroporated at 400 V for 1 s [21,41]. No oligonucleotide controls were processed identically. Thereafter, the samples were incubated in the optimum environment for 0 h, 5 h, 10 h, 15 h, and 20 h. RNA was extracted from samples at different time points and qPCR was performed as detailed in Section 4.3.1. The appearance of the sample was observed using a microscope.

### 4.9. Bioinformatics

Using the 3′ RACE and 5′ RACE techniques, the sequence of full-length *pdrg1* cDNA of *Artemia* was obtained using DNAman 8.0 software. The nucleic acid sequence of the *Artemia pdrg1* gene was submitted to NCBI to compare the amino acid of BLASTX (available online: http://blast.ncbi.nlm.nih.gov/Blast/) to determine its homology and domains. The ORF in the *pdrg1* cDNA was determined using ORF FINDER (https://www.ncbi.nlm.nih.gov/orffinder/) and InterProscan software (http://www.ebi.ac.uk/Tools/pfa/iprscan/) was used to analyze the domain and conserved sites in the predicted protein. The theoretical molecular weight and isoelectric point of PDRG1 were deduced by ProtParam (http://web.expasy.org/protparam/). The hydrophobicity and transmembrane regions of the protein were analyzed using Protscale (http://web.expasy.org/protscale/) and TMHMM software, respectively. The subcellular localization, phosphorylation sites, and signal peptide analysis of the PDRG1 protein were predicted using Psort II (http://psort.hgc.jp/form2.html), NetPhos 3.1 (http://www.cbs.dtu.dk /services/NetPhos/), and SignalP 4.1 (http: //www.cbs.dtu.Dk/services/SignalP/), respectively. The secondary structure of the protein was analyzed using the SOPM method (https://npsa-prabi.ibcp.fr/cgi-bin/npsa_automat.pl?page=/NPSA/npsa_server.html). The phylogenetic analysis of the *pdrg1* gene was carried out using Clustal X and MEGA 6.0. The neighbor-joining (NJ) method was used to construct the phylogenetic tree. The Bootstrap method was used to evaluate the accuracy of the tree (Bootstrap = 1000). The statistical significance of any change was analyzed using the least square difference (LSD) *t*-test in SPSS 16.0. The significance threshold was *p* < 0.05.

## 5. Conclusions

The full-length cDNA of the *pdrg1* gene (*As-pdrg1*) was obtained from *A. sinica* and found to contain 1119 nucleotides, including a 228 bp open reading frame (ORF), which encodes a 75 amino acid protein. We determined that the increased mRNA and protein expression levels of *As*-PDRG1 could promote apoptosis to remove unwanted, damaged, and dangerous cells during development and to maintain homeostasis [19]. These results suggested that *As*-PDRG1 plays an important role in diapause termination and early embryonic development of *A. sinica*. To study the function *As*-PDRG1, p53 and related proteins (MDM2, p21) expression was analyzed by Western blotting. Although *As-pdrg1* expression was decreased by p53 negative regulation, the *As*-PDRG1 showed high expression at 20 h and 40 h. The increased expression occurred in a p53 non-dependent manner. It is possible that strong positive regulation by other genes could override the negative effect of p53 on PDRG1. This phenomenon is similar to the result of Luo [16]. In their study, the *pdrg1* gene promoter harbors Oct1-binding sites, and Oct1 strongly upregulates PDRG1 expression following genotoxic stress. Since the *pdrg1* has functions such as DNA damage repair, we examined the expression of *As-pdrg1* under low temperature. The result showed that *As-pdrg1* is highly expressed under low temperature, which confirmed that *As-pdrg1* is likely to be involved in DNA damage repair. However, at the protein level, this phenomenon was not obvious. This might be caused by more complex regulatory mechanisms involving *As*-*pdrg1*, resulting changes in its expression and inconsistent RNA levels. This mechanism remains unclear requiring further research. The results of siRNA knockdown of *As-pdrg1* suggested that it is indispensable for the growth and development of *A. sinica.* This study provided indirect evidence for the potential functions of PDRG1 proteins in animals and a molecular mechanism of the stress tolerance exerted by PDRG1 proteins in *A. sinica*. In conclusion, *As*-PDRG1 is strictly required for the processes of diapause embryo resumption and early embryo development in *A. sinica*.

## Figures and Tables

**Table 1:** Predicted phosphorylation sites in *As*-PFRG1.

Name	Position	Context ^a^	Score ^b^
Ser	5	MRFGSSMFK	0.500
	6	RFGSSMFKI	0.841
	40	ILKDSVMVL	0.675
	45	VMVLSQIED	0.941
	50	QIEDSPGMK	0.950
	59	GLNLSPLGS	0.802
	63	SPLGSEERK	0.812
	70	RKAISDLIG	0.578

^a^ Sequences surrounding the phosphorylation sites. ^b^ Probability of the phosphorylation sites.

**Table 2:** Oligonucleotide primers used in this study.

Primer	Sequence (5′-3′)	Direction
*As-pdrg1*F	CTGAAATAGAAGAGGCAGGT	Forward
*As-pdrg1*R	CTATTTCGGATTTGATTGGT	Reverse
3′*As-pdrg1*	TGAGCCCATTGGGTAGTGAAGA	Forward
5′*As-pdrg1*	TGAACAGGAGATAAATTTCATAG	Reverse
ISH-*pdrg1*F	TGAACAGGAGATAAATTTCATAGCTG	Forward
ISH-*pdrg1*R	TTGATTGGTGATTAAGGTGTTGAAA	Reverse
RT-*pdrg1*F	TGGTGAAGACGAATAGGTCCA	Forward
RT-*pdrg1*R	AAGTGCAGTGCAAGCTAACC	Reverse
*β*-actin-F	GTGTGACGATGATGTTGCGG	Forward
*β*-actin-R	GCTGTCCTTTTGACCCATTCC	Reverse
ORF-*pdrg1*F	TGAACAGGAGATAAATTTCATAG	Forward
ORF-*pdrg1*R	TTTCAACACCTTAATCACCAATC	Reverse
Sense siRNA	GGAUUUGAUUGGUGAUUAAUU	Forward
Anti siRNA	UUCCUAAACUAACCACUAAUU	Reverse

## Abbreviations

PDRG1	p53 and DNA damage-regulated gene 1
*As-pdrg1*	mRNA from *Artemia sinica*
*As*-PDRG1	protein from *Artemia sinica*
ORF	open reading frame
PCR	polymerase chain reaction
UTR	untranslated region
ISH	in situ hybridization
siRNA	short interfering RNA
DIG	digoxigenin

## References

[1] Zhang S., Yao F., Jing T., Zhang M., Zhao W., Zou X., Sui L., Hou L. (2017). Cloning, expression pattern, and potential role of apoptosis inhibitor 5 in the MARK termination of embryonic diapause and early embryo development of *Artemia sinica*. Gene.

[2] Zhang M.C., Yao F., Luan H., Zhao W., Jing T., Zhang S., Hou L., Zou X.Y. (2016). APC/CCDC20 and APC/C play pivotal roles in the process of embryonic development in *Artemina sinica*. Sci. Rep..

[3] Abatzopoulos T.J., Beardmore J.A., Clegg J.S., Sorgeloos P. (2002). Artemia: Basic and Applied Biology.

[4] Li X., Hou L., Ma J., Liu Y.D., Zheng L.P., Zou X.Y. (2012). Cloning and character-ization of β-catenin gene in early embryonic developmental stage of *Artemia sinica*. Mol. Biol. Rep..

[5] Hou L., Jiang L.J., Sun W.J., Zhang R.F., Wang J.Q., Zhao X.T., An J.L. (2006). Establishment and improvement of real-time fluorescence quantitative PCR for actin gene of *Artemia sinica*. J. Liaoning Normal Univ..

[6] Wang X.L., Yao F., Liang X.Y., Zhu X.L., Zheng R., Jia B.L., Hou L., Zou X.Y. (2016). Cloning and expression of retinoblastoma-binding protein 4 gene in embryo diapause termination and in response to salinity stress from brine shrimp *Artemia sinica*. Gene.

[7] Jiang L.J., Hou L., Zou X.Y., Zhang R.F., Wang J.Q., Sun W.J., Zhao X.T., An J.L. (2007). Cloning and expression analysis of p26 gene in *Artemia sinica*. Acta Biochim. Biophys. Sin..

[8] .Zheng L.P., Hou L., Chang A.K., Yu M., Ma J., Li X., Zou X.Y. (2011). Expression pattern of a gram-negative bacteria-binding protein in early embryonic development of *Artemia sinica* and after bacterial challenge. Dev. Comp. Immunol..

[9] Li Z., Yao F., Chen Y., Zhang R., Lv Y., Zhao N., Wang T., Xin W., Hou L., Zou X. (2013). Molecular cloning, characterization and expression analysis of ubiquitin pro-tein ligase gene (*As*-*ubpl*) from *Artemia sinica*. Mol. Cell Biol..

[10] Qing T., Zhao X., Luan H., Ba H., Yang L., Li Z., Hou L., Zou X. (2015). Identification, expression pattern and functional characterization of *As*-MyD88 in bacteria challenge and during different developmental stages of *Artemia sinica*. Dev. Comp. Immunol..

[11] Zhao W., Yao F., Zhang M.C., Jing T., Zhang S., Hou L., Zou X.Y. (2016). The Potential Roles of the G1LEA and G3LEA Proteins in Early Embryo Development and in Response to Low Temperature and High Salinity in *Artemia sinica*. PLoS ONE.

[12] Sardiu M.E., Cai Y., Jin J., Swanson S.K., Conaway R.C., Conaway J.W., Florens L., Washburn M.P. (2008). Probabilistic assembly of human protein interaction networks from label-free quantitative proteomics. Proc. Natl. Acad. Sci. USA.

[13] Boulon S., Bertrand E., Pradet-Balade B. (2012). HSP90 and the R2TP co-chaperone complex: Building multi-protein machineries essential for cell growth and gene expression. RNA Biol..

[14] Mita P., Savas J.N., Ha S., Djouder N., Yates J.R., Logan S.K. (2013). Analysis of URI nuclear interaction with RPB5 and components of the R2TP/prefoldin-like complex. PLoS ONE.

[15] Wang T., Yang H. (2013). Cloning and characterization of PDRG gene from sea cucumber Apostichopus japonicus and the expression in intestineduring aestivation. Mar. Sci..

[16] Luo X.Q., Huang Y., Sheikh M.S. (2003). Cloning and characterization of a novel gene PDRG that is differential lyregulated by p53 and ultraviolet radiation. Oncogene.

[17] Jiang L., Luo X., Shi J., Sun H., Sun Q., Sheikh M.S., Huang Y. (2011). PDRG1, a novel tumor marker for multiple malignancies that is selectively regulated by genotoxic stress. Cancer Biol. Ther..

[18] Gong W.L., Xu G., Han Q.Y., Chen Y. (2013). Construction of HeLa-PDRG1-knock Down Stable Cell Line by Lentivirus Infection. Sci. Technol. Eng..

[19] Pérez C., Pérez-Zúñiga F.J., Garrido F., Reytor E., Portillo F., Pajares M.A. (2016). The Oncogene PDRG1 Is an Interaction Target of Methionine Adenosyltransferases. PLoS ONE.

[20] Zhao C., Dai W., Qiu L. (2017). Molecular cloning, characterization and expression analysis of a novel PDRG1 gene from black tiger shrimp (*Penaeus monodon*). Genet. Mol. Biol..

[21] Chu B., Yao F., Cheng C., Wu Y., Mei Y.L., Li X.J., Liu Y., Wang P.S., Hou L., Zou X.Y. (2014). The potential role of As-sumo-1 in the embryonic diapause process and early embryo development of *Artemia sinica*. PLoS ONE.

[22] Zhang Q.Z., Hou M., Li Q.Y., Han L.L., Yuan Z., Tan J., Du B., Zou X.Y., Hou L. (2013). Expression patterns of As-ClC gene of *Artemia sinica* in early development and under salinity stress. Mol. Biol. Rep..

[23] Goh A.M., Xue Y., Leushacke M., Li L., Wong J.S., Chiam P.C., Rahmat S.A., Mann M.B., Mann K.M., Barker N. (2015). Mutant p53 accumulates in cycling and proliferating cells in the normal tissues of p53 R172H mutant mice. Oncotarget.

[24] Faried A., Faried L.S., Kato H., Asao T., Kuwano H., Yazawa S. (2008). Targeting P53 tumor suppressor to induce apoptosis and cell cycle arrest in esophageal cancer cells by novel sugar-cholestanols compounds. Poster Sess..

[25] Melino G., Lu X., Gasco M., Crook T., Knight R.A. (2003). Functional regulation of p73 and p63 development and cancer. Trends Biochem. Sci..

[26] Liu D., Zhang J., Wu Y., Shi G., Yuan H., Lu Z., Zhu Q., Wu P., Lu C., Guo F. (2017). YY1 suppresses proliferation and migration of pancreatic ductal adenocarcinoma by regulating the CDKN3/MdM2/P53/P21 signaling pathway. Int. J. Cancer.

[27] Ma J., Li Y., Wu M., Li X. (2017). Oxidative stress-mediated p53/p21WAF1/CIP1 pathway may be involved in microcystin-LR-induced cytotoxicity in HepG2 cells. Chemosphere.

[28] Li X.R., Lin X.Y., Xiao Z.X., Jiang H.X., Tang Q.L., Liu X. (2008). Structure and Function of P21 and New Research Progress. Med. Recapitul..

[29] Luo J.L., Cao J.P., Zhu W., Feng S., Sheng F.J., Zhu C.Y., Zheng S.Y. (2005). Interaction between ATM and Radiation-activated Phosphorylation of P53 and P21. Chin. J. Cancer.

[30] Cheng C., Yao F., Chu B., Li X.J., Liu Y., Wu Y., Mei Y.L., Wang P.S., Hou L., Zou X.Y. (2014). Identification of the glycerol kinase gene and its role in diapause embryo restart and early embryo development of *Artemia sinica*. Gene.

[31] Koci L., Chlebova K., Hyzdalova M., Hofmanova J., Jira M., Kysela P., Kozubik A., Kala Z., Krejci P. (2012). Apoptosis inhibitor 5 (API-5; AAC-11; FIF) is upregulated in human carcinomas in vivo. Oncol. Lett..

[32] Saigusa S., Tanaka K., Toiyama Y., Matsushita K., Kawamura M., Okugawa Y., Hiro J., Inoue Y., Uchida K., Mohri Y. (2012). Gene expression profiles of tumor regression grade in locally advanced rectal cancer after neoadjuvant chemoradiotherapy. Oncol. Rep..

[33] Sorgeloos P., Lavens P., Leger P., Tackaert W., Versuchele D. (1986). Manual for the Culture and Use of Brine Shrimp Artemia in Aquaculture.

[34] Hallstrom T.C., Mori S., Nevins J.R. (2008). An E2F1-dependent gene expression pro-gram that determines the balance between proliferation and cell death. Cancer Cell.

[35] Schmittgen T.D., Livak K.J. (2008). Analyzing real-time PCR data by the comparative CT method. Nat. Protoc..

[36] Bradford M.M. (1976). A rapid and sensitive method for the quantitation of microgram quantities of protein utilizing the principle of protein–dye binding. Anal. Biochem..

[37] Livak K.J., Schmittgen T.D. (2001). Analysis of relative geneexpression data using real-time quantitative PCR andthe 2(-Delta Delta C(T)) method. Methods.

[38] Verger A., Perdomo J., Crossley M. (2003). Modification with SUMO A role in transcriptional regulation. EMBO Rep..

[39] Azmi T.I., Oshea J.D. (1984). Mechanism of deletion of Endo-thelial-Cells during regression of the Corpus-Luteum. Lab. Investig..

[40] Erica S.J., Gunter B. (1997). Ubc9p Is the Conjugating Enzyme for the Ubiquitin-like Protein Smt3p. J. Biol. Chem..

[41] Freichel M., Vennekens R., Olausson J., Stolz S., Philipp S.E., Weissgerber P., Flockerzi V. (2005). Functional role of TRPC proteins in native systems: Implications from knockout and knock-down studies. J. Physiol..

